# Supplementary material for: Are carbon emissions trading and green financial instruments synergistic? -Comprehensive quantitative research based on content analysis
Source: PLoS One. 2024 Mar 7;19(3):e0298601. doi: 10.1371/journal.pone.0298601 (PMC10919601; doi:10.1371/journal.pone.0298601)
Supplement: S1 Data — (ZIP) [file pone.0298601.s001.zip › Data/地级市Z_F工作报告.zip!/║■─╧/╥μ╤⌠╩╨/└┤╫╘╣1⁄2╓┌║┼í╛┬φ┐╦╩2╛▌═°í┐_2019.docx]

各位代表：

现在，我代表市人民政府向大会作工作报告，请予审议，并请各位政协委员和其他列席人员提出意见。

2018年工作回顾

2018年，是改革开放40周年，是我市经济社会发展历程中不平凡的一年。一年来，我们坚持以习近平新时代中国特色社会主义思想为根本遵循，全面贯彻习近平总书记对湖南工作的重要指示精神，坚决落实省委、省政府和市委重大决策部署，突出高质量发展要求，紧扣“六稳”工作重点，以“五先五市”为总抓手，聚焦聚力稳增长、促改革、调结构、惠民生、防风险，总体完成年度目标任务。全市实现生产总值1782亿元（预计数，下同），比上年增长8.2%；固定资产投资增长11%；规模工业增加值增长8%；社会消费品零售总额增长10.3%；完成一般公共预算收入125.8亿元，同口径增长8.5%；全体居民人均可支配收入增长9.2%。经济社会发展保持稳中有进、进中向好的良好态势。

——脱贫攻坚取得阶段性胜利，全市贫困发生率降到1.35%，安化县通过贫困县摘帽省级实地检查验收。

——常益长高铁建设顺利推进，马安高速提前一年建成通车，全市高速公路通车里程达360公里，中心城区绿色公交全面实现。

——工业经济增速明显加快，规模工业增加值增速自5月份以来连续8个月领跑全省。

——现代农业“131千亿级产业”工程亮点纷呈，茶产业综合产值增长20%，稻虾等生态种养面积达78万亩，每亩平均增收3000元以上。南洲稻虾米进入香港市场。

——宇晶股份成功上市，全市主板上市企业达6家，居全省第4位。

——“益山益水·益美益阳”品牌影响力持续增强，成功举办第二届生态农业智慧乡村互联网大会、第二届“汇智聚力·建设益阳”恳谈会、第十届中国竹文化节、第四届黑茶文化节、第二届涂鸦艺术节、第二届洞庭湖生态文化旅游节。

——新型智慧城市建设积极推进，荣获“2018年中国城市治理智慧化优秀奖”“互联网+政务服务”走在全省前列。

——益阳海关获批成立，发展开放型经济取得突破性进展。

——成功创建全国环境空气质量达标城市。

——消除大班额1238个，其中超大班额922个，超额完成省定目标任务。

一年来，我们突出抓好六个方面工作：

一、着力打好三大攻坚战

防范化解重大风险成效明显。严格落实省委、省政府“停缓调撤”要求，全面清理政府性投资项目，多渠道筹集资金还本付息，平台公司转型平稳推进，政府债务风险总体可控。市本级财政绩效综合管理连续两年获得全省最高评分。加大处非工作宣传和隐患排查力度，妥善处置4起非法集资存案，守住了不发生区域性金融风险底线。精准脱贫实现既定目标。制定实施产业兴市暨产业扶贫三年行动计划，扎实开展“脱贫攻坚百日会战”，“六个一批”“六大建设”有力推进。全市投入脱贫攻坚资金20.6亿元，建成51个易地扶贫搬迁项目，完成自然村通水泥（沥青）路1445公里，8.2万人实现安全饮水。10.08万贫困人口脱贫，122个贫困村出列。污染防治取得重大进展。落实“共抓大保护，不搞大开发”要求，以河长制湖长制为总揽，以“一江两湖”、6大片区水环境治理为重点，统筹推进洞庭湖生态环境整治、资江流域锑污染防治、大气污染防治、农村人居环境整治4个三年行动计划，着力打好碧水、蓝天、净土保卫战。狠抓中央、省环保督察反馈问题整改和总河长令落实，彻底解决下塞湖矮围问题，完成中央环保督察反馈问题整改11个、信访办结153件；办理省环保督察反馈问题29个、信访办结400件。饮用水源和中心城区规划区山体水体保护、黑臭水体治理、黑杨清理、黏土砖厂和“两围”整治、造纸企业引导退出等重点工作全面推进。资江益阳段总体水质为优，大通湖水质逐步改善，中心城区环境空气质量综合指数明显提升。

二、着力抓好产业建设

“产业项目建设年”活动扎实开展。全力实施省“5个100”和市“6个10”工程。新开工项目242个，新投产项目216个，奥士康第三科学园、艾华二期、佳纳能源等项目顺利开工，顺祥水产二期、口味王赫山基地、芙蓉云数据中心、软通动力、福德电气二期等项目投产运营。新型工业化快速发展。全力推进十大工业新兴优势产业链建设，深入开展企业帮扶、品牌建设和质量提升行动，着力提高工业发展质效。规模工业企业新增127家，总数达到1132家。工业用电量增长14.2%，增速居全省第二。园区规模工业增加值增长8.5%，新增标准化厂房115.8万平方米。现代农业稳步发展。突出抓好国家现代农业示范区和省现代农业改革试验市建设，实现农林牧渔业总产值450.8亿元，增长3.5%，增速稳居全省前列。新增“三品一标”认证农产品65个，赫山兰溪大米等5个农产品获国家级登记认证。安化黑茶产业园和桃江楠竹产业示范园获批国家级称号，南县被评为“中国虾稻米之乡”。新型农业经营主体健康发展，农民合作社和家庭农场分别新增950家、586家，省级以上星级休闲农庄达67家。有力防控非洲猪瘟，“大棚房”清理整治工作有序推进。现代服务业蓬勃发展。天意木国、凤凰酒店等重点旅游项目建成开业。安化黑茶小镇、茶乡花海、桃花江旅游度假区建设顺利推进。全市接待游客人次、旅游收入分别增长29.7%、35%。加大信贷投放力度，年末金融机构存、贷款余额分别为1930亿元和1065亿元，新增贷款152亿元，存贷比提升5.4个百分点。电子商务加快发展，实现线上交易额550亿元，中南电商园获评第二批国家电子商务示范基地，快递进出港业务量突破1亿件，增长39.1%。房地产业健康发展，投资和商品房销售面积分别增长10%、5%。

三、着力加强城乡建设

基础设施不断完善。益马、马安高速公路和G234南县至茅草街等12条干线公路建成通车，S324东坪至梅城等11个项目有序推进。新建和改扩建农村公路2104公里，行政村全部开通客班车。常益长高铁启动征地拆迁，完成高铁站综合交通枢纽规划编制。水利冬春修完成投资34.1亿元，整修各类水利工程4万余处，完成33座水库除险加固，五七闸补水工程建成启用，中央财政农田水利建设项目扎实推进，防汛抗旱实现“五个确保”目标。500千伏益阳东变电站等5项重点输变电工程顺利实施，（下转第2版）

（上接第1版）357个自然村完成农网升级改造。益阳电厂分布式光伏发电等4个新能源项目并网发电。华容－南县－安乡天然气管道竣工投产，长沙－益阳天然气管道一期主体工程完成。行政村光网和4G覆盖率达100%。城乡建设加快推进。《益沅桃城镇群城镇体系规划（2016－2030年）》获批。国家森林城市复查顺利通过，西流湾大桥建成通车，实施“三微”二期项目346个，梅林路建设及竹山湾片区排水改造工程如期完成。智慧教育、智慧城管、雪亮工程等新型智慧城市重点项目稳步推进。制定《益阳市中心城区资江风貌带管理暂行办法》，“铁锤行动”拆除违建73万平方米，中心城区货车禁限行、户外广告整治成效明显。县城和重点镇建设步伐加快，出台《益阳市农村村民住房建设管理条例》，创建县级以上美丽乡村示范村129个，获批中国传统村落5个，城乡规划建设管理水平有效提升。

四、着力推进改革开放

各项改革不断深化。新一轮机构改革顺利推进，83项改革事项稳步实施。深入推进“放管服”改革，市本级行政许可事项压减至106项，向园区下放90项、向东部新区下放29项市级经济管理权限。农村土地确权颁证通过省级验收，增量配电改革积极推进。创新活力明显增强。全市高新技术企业达到160家，高新技术产业增加值占生产总值比重达18.5%，全社会研发投入占生产总值比重达1.48%，每万人拥有有效发明专利2.3件。荣获第十届国际发明展览会金奖8项，创历史最佳成绩。新增国家、行业、地方标准18项，企业标准426项。对外开放取得突破。与孟加拉国拉杰沙希市缔结友好城市。节会签约项目117个，合同引资396亿元，签约落地500强企业投资项目14个。引进市域外资金形成固定资产投资578亿元，增长15%。实现进出口总额11亿美元，增长39.2%，获评“全省发展开放型经济优秀单位”。

五、着力增进民生福祉

民生保障更加有力。12项重点民生实事全面完成。新增市场主体2.7万个、城镇就业3.4万人、农村劳动力转移就业1.7万人，城镇登记失业率控制在3.5%以内。十大创新创业活动深入开展，青年创新创业孵化基地挂牌运行。五大社保参保870万人次，实现基本养老保险、基本医疗保险全覆盖。完成城镇棚户区改造2.25万套、农村危房改造1.6万户。残疾人关爱、社会救助、移民避险解困等工作统筹推进。社会事业全面进步。推进教育强市建设，新建、改扩建学校77所，培养公费定向师范生820名。职业教育取得新进展，船舶工程技术和健康养老服务专业列入省一流特色专业群项目。湖南城市学院获批省应用特色学科6个。30位教育工作者被授予第二届教育突出贡献奖。深化医药卫生体制改革，医联体和专科联盟建设取得实质性进展，41家涉改公立医院全面实施药品零差率销售，传染病疫情得到有力控制，基本公共卫生和计划生育综合服务措施有效落实。农村贫困患者实行先诊疗后付费和“一站式”结算。市妇幼保健院搬迁项目主体工程建成，市残疾人康复中心建设顺利推进。荣获全国无偿献血先进市和省医养结合模式创新奖。加快文化体育事业发展，举办第七届花鼓戏汇演，公共文化体系建设取得新进展，积极创建全民运动健身模范市，承办全国羽毛球冠军赛等重大赛事，在第六届湖南艺术节和十三届省运会上取得良好成绩。社会治理持续深化。开展扫黑除恶专项斗争，破获一批涉黑涉恶案件，抓捕一批犯罪嫌疑人，形成有效震慑，人民群众拍手称快。三年禁毒大行动取得成效，平安益阳创建深入推进。全面实施打非治违“百日行动”和安全监管“强执法防事故”行动，安全生产形势持续稳定。公路“治超”取得实效，道路安全管理力度加大，交通安全形势明显改善。扎实开展“信访法治建设年”活动和涉访“四大攻坚”。公共法律服务体系不断完善，办理法律援助案件2408件。精神文明建设不断加强。实施社会主义核心价值观引领工程，全力推进全国文明城市创建，志愿者行动深入开展，群众参与度和获得感明显提升，城市文明程度不断提高。引导破除陈规陋习，积极推动殡葬改革，戒奢崇俭新风尚逐步形成。

六、着力强化自身建设

政务服务进一步提效。“互联网+政务服务”强力推进，被确定为全省创新示范市。市、县一体化平台建成运行，审批服务便民化取得实效，申请类事项网上可办率达98%，办理时限平均压缩50%以上。权力运行进一步规范。主动接受人大及其常委会法律监督、工作监督和政协民主监督，积极落实人大及其常委会决议决定和审议意见，办理人大代表建议201件、政协委员提案236件，办理质量明显提升。坚持政府常务会议会前学法制度，完善重大行政决策程序，严格落实行政执法监督暂行办法，依法行政水平不断提高。廉政建设进一步加强。落实全面从严治党要求，推进“两学一做”学习教育常态化制度化。贯彻落实中央八项规定及实施细则，驰而不息纠“四风”。强化党风廉政建设“一岗双责”，加强重点领域、重点环节廉政风险防控，严肃查处一批民生保障、脱贫攻坚、环境保护、项目建设、公共资源交易等领域违法违纪案件，政风行风进一步好转。

国防动员、武警、消防、人防和军民融合等工作不断加强。工商行政管理、质量技术监督和食品药品监管不断强化。第四次经济普查扎实推进。供销合作、民族宗教、住房公积金、公共资源管理、科协、档案、保密、对台、地震、气象、水文、地方志、红十字会、援疆等工作不断进步。大力弘扬劳模精神和工匠精神，表彰100名劳动模范、先进工作者和益阳工匠，工会、共青团、妇联等群团组织在经济社会发展中发挥了积极作用。

各位代表，2018年是我们团结拼搏、励精图治的一年，也是我们攻坚克难、砥砺前行的一年。2018年的这份答卷，是在应对经济下行复杂形势、顶住风险隐患增多重重压力、克服各种现实困难情况下完成的，实属来之不易，值得倍加珍惜。这些成绩的取得，是以习近平同志为核心的党中央坚强领导的结果，是省委、省政府和市委正确领导的结果，是市人大、市政协监督支持的结果，是全市上下共同努力的结果。在此，我谨代表市人民政府，向全市人民，向各民主党派、工商联、无党派人士、各人民团体，向驻益人民解放军和武警官兵、政法干警，向中央、省属驻益单位，向热心支持益阳经济社会发展的社会各界人士，表示衷心感谢并致以崇高敬意！

在总结成绩的同时，我们也清醒认识到，发展中仍有不少困难和问题，主要是：经济下行压力加大，新旧动能转换不快；经济总量不大，产业结构不优，发展质效与高质量发展要求还有较大差距；企业生产经营困难较多，融资难、融资贵等问题尚未得到有效缓解；政府性债务进入还本付息高峰期，风险防控任务仍然繁重；环境污染防治任务仍然艰巨；民生保障水平有待进一步提高；少数干部发展观念、工作作风、精神状态与创新开放要求不相适应，等等。我们必须增强忧患意识，抓住主要矛盾，采取有力措施，切实加以解决。

2019年工作安排

2019年，是新中国成立70周年，是决胜全面建成小康社会第一个百年奋斗目标的关键一年。2019年政府工作的指导思想是：以习近平新时代中国特色社会主义思想为指导，坚持稳中求进工作总基调，坚持新发展理念，坚持推动高质量发展，坚持以供给侧结构性改革为主线，坚持深化市场化改革、扩大高水平开放，坚持创新引领开放崛起，坚持“五先五市”总抓手，继续打好三大攻坚战，持续推进“产业项目建设年”活动，聚力先进制造业、高技术产业和园区经济，全力稳就业、稳金融、稳外贸、稳外资、稳投资、稳预期，保持定力、提振信心、担当作为、奋发进取，保持经济持续健康发展和社会大局稳定，为全面建成小康社会收官打下决定性基础，以优异成绩向中华人民共和国成立70周年献礼。

2019年经济社会发展的主要目标是：生产总值增长8%左右，固定资产投资增长11.5%，规模工业增加值增长8%左右，社会消费品零售总额增长10.5%，一般公共预算收入增长7.5%，全体居民人均可支配收入增速与经济增长基本同步。

按照高质量发展要求，更加注重以下指标：税收占一般公共预算收入比重达到82.5%以上；高新技术产业增加值占生产总值比重达到18.8%以上；研发经费支出占生产总值比重达到1.92%；制造业投资占固定资产投资比重达到37%；单位生产总值能耗下降3%；环境空气质量优良天数比例达到90%以上；文化及相关产业增加值占生产总值比重达到4%；城镇登记失业率控制在省定目标以内。

完成上述目标任务，必须切实抓好以下八个方面的工作：

一、践行新发展理念，奋力打好三大攻坚战

突出打好防范化解重大风险、精准脱贫、污染防治三大攻坚战重点战役，为高质量发展扫清障碍、腾出空间、夯实基础。坚决守住不发生重大风险底线。贯彻落实《中共中央国务院关于防范化解地方政府隐性债务风险的意见》，妥善防控政府债务风险，切实做到坚定、可控、有序、适度。执行负面清单管理，严格规范PPP与政府购买服务项目。加快推进平台公司转型发展。积极创建金融安全区，强化薄弱环节监管，严厉打击非法集资等金融违法犯罪行为，优化金融生态环境，守住不发生区域性风险和大规模群体性事件两条底线。坚决完成脱贫攻坚年度任务。强化责任、工作、政策“三落实”，巩固脱贫成果，突出产业扶贫，继续实施产业兴市暨产业扶贫三年行动计划，深入开展“万企帮万村”“互联网＋社会扶贫”工作，动员全社会参与脱贫攻坚。加强扶贫领域资金监管，提高扶贫资金使用效益。确保3.5万贫困人口脱贫、34个贫困村出列。坚决整治污染突出问题。以中央和省环保督察反馈问题整改为重点，继续打好碧水、蓝天、净土保卫战。夯实河长制湖长制工作基础，巩固提升“三长一站”工作机制，强化四级河长责任。深入推进洞庭湖生态环境整治和资江流域锑污染防治三年行动计划，实施10大领域和6大片区集中治理，着力抓好大通湖流域治理试点、饮用水源保护、入河口排污和非法采砂整治、湿地修复及造纸产能引导退出等重点工作。继续实施大气污染防治三年行动计划，完善防治体系，巩固提升全国环境空气质量达标城市创建成果。加快光大环保二期、西部和北部生活垃圾焚烧发电、餐厨垃圾和固体废弃物资源化利用等项目建设，新建16个乡镇污水处理厂。推进城乡黑臭水体整治，中心城区建成区消除比例达90%以上，县城和农村完成省定整治目标任务。确保生态环境质量持续改善。

二、聚焦高质量发展，加速推进新型工业化

推动工业经济加快发展，工业投资增长10%以上，新增规模工业企业100家以上。发展壮大优势产业。深入实施十大工业新兴优势产业链推进方案，围绕食品加工、装备制造、电子信息、生物医药、新材料等特色优势产业，重点在延链、补链、强链上下功夫。继续开展“产业项目建设年”活动，推进省“5个100”和市“6个10”重大产业项目建设。注重引进一批重大科技创新项目，着力实施一批重大技改项目，推动奥士康第三科学园、艾华二期、中联重科沅江智能制造工业园、佳纳能源和湘易康等重点项目加快建设。做好益阳电厂三期扩建等重大项目前期工作。加快发展数字经济。突出抓好“一中心三基地”建设，着力引进一批移动互联网、云计算、大数据、物联网和人工智能企业落户益阳，在产业数字化和数字产业化上迈出坚实步伐。继续推进园区建设大会战。抓好向园区下放市级经济管理权限决定的落实，推进“园区事园区办”和“一站式服务”，支持园区聚焦聚力招商引资和产业建设，减轻园区社会事务负担，防止园区出现行政化倾向。推动益阳高新区加快发展、做大做强，发挥国家级高新区引领带动作用。强化规划引领，完善公共服务配套，提升园区承载能力，提高园区单位产出和税收贡献。按照打造“135”工程升级版要求，加快军民融合“一园两基地”等特色园区建设，新增园区固定资产投资100亿元以上，新开工、新投产工业项目各100个以上。着力发展实体经济。贯彻落实促进民营经济发展相关政策，降低企业生产经营成本。持续开展企业精准帮扶行动，加大金融支持实体经济力度，将政银企对接活动贯穿全年，深入开展金融服务进园区进企业、促融资促发展“两进两促”主题活动。充分发挥市融资担保公司作用，新增担保业务10亿元以上，努力缓解企业融资难、融资贵问题。规范涉企执法行为，切实保护民营企业合法权益。继续开展质量提升行动，加强企业品牌建设。实施中小企业“小巨人”培育计划，培育壮大一批“专精特新”企业。坚持把企业上市作为发展实体经济、推动产业转型升级的战略性举措，支持条件成熟的企业上市融资加快发展。

三、实施乡村振兴战略，扎实推进农业农村现代化

实施乡村振兴战略规划，加快推动我市由农业大市向农业强市转变。打造优质农副产品供应基地。持续推进现代农业“131千亿级产业”工程，大力发展特色产业，打造区域公用品牌。落实粮食生产责任制，推进高标准农田建设，稳定粮食生产。优化种养结构，提升产出效益，推动稻田综合种养面积向100万亩进军。积极推进南县、赫山、沅江“中国好粮油”行动示范县建设。加强农产品质量安全监管，抓好重大动植物疫病防控，推进标准化、绿色化、规模化生产，新增“三品一标”认证农产品50个。推动农业转型升级发展。促进一二三产业有机融合，大力发展农产品精深加工，重点扶持农业产业化龙头企业，探索开展田园综合体建设，精心打造体验农业、创意农业、观光农业等新型业态。推进农村土地规范有序流转，引导土地适度规模经营。继续实施新型职业农民培育工程，支持新型农业经营主体扩容提质，新增市级示范家庭农场和农民合作示范社各20家。强化农业科技支撑。加强农业科技人才引进和培育，推动农业实用技术科研攻关，加快现代农业产业园、科技园、创业园提质升级。加强农机农艺融合，落实农机购置补贴政策，提升农业机械化水平。建好智慧农业平台，加快农业现代信息技术推广应用，深入推进“互联网+现代农业”。推进美丽乡村建设。因地制宜、因村制宜，科学编制村庄规划。推进农村人居环境整治三年行动计划和“百村示范、千村整治”工程，重点抓好厕所革命、农业面源污染、农村生活垃圾污水治理等工作。开展国土绿化行动，实施防护林建设、石漠化治理等重点工程，提升村庄绿化美化水平。新增县级以上美丽乡村示范村30个。加强乡村文明建设。完善自治、法治、德治相结合的乡村治理体系和农村公共文化服务体系，加快补齐农村基础设施和基本公共服务短板。扩大禁放范围，推进殡葬改革，狠刹人情歪风，持续发力破除陈规陋习，推动移风易俗，引导建设良好家风，培育文明乡风、淳朴民风。

四、培育新兴业态，着力发展现代服务业

坚持生产性服务业与生活性服务业并重，新兴服务业与传统服务业并举，加快构建现代服务业体系。深入推进全域旅游。积极发展乡村旅游、休闲旅游、假日旅游，建设一批旅游精品线路和特色景区。推动旅游与文化、康养、体育、研学等融合发展。加快重点旅游项目建设，完善旅游服务配套设施，支持区县（市）举办特色旅游营销活动，大力推介特色餐饮。着力打造“休闲益阳”。加快发展现代金融。引进股份制商业银行，推进非银行类金融机构建设，完善融资担保体系。充分发挥保险补偿稳定功能，扩大农业保险等政策性保险覆盖范围，切实提高保险密度和深度。创新金融产品，优化金融服务，扩大信贷规模，新增贷款150亿元以上。积极发展商贸物流。稳步推进城市综合体建设，增强万海商圈辐射带动效应。实施商贸流通“千百工程”，培育新增限额以上商贸流通企业100家。支持传统服务业标准化建设，积极打造零售、批发、餐饮、酒店、电商和流通综合体六类“十强企业”。加强农村物流基础设施建设，加快电子商务进农村，依托益村平台和供销e家平台，推动县级电商运营中心向乡村延伸，完善县乡村三级快递配送体系，打通农村电商“最后一公里”。大力发展康养产业。编制康养产业发展规划，完善政策支持体系。积极发展集医疗护理、康复保健、生命关怀和旅游度假于一体的康养模式，加快建设医养融合型社会服务机构，鼓励社会资本进入养老服务市场。抓好全国居家和社区养老服务改革试点工作，提升康养服务质效。推动房地产业健康发展。加强市场研判，完善房价联合管控机制。妥善处置问题楼盘，进一步规范二手房交易和房屋租赁市场，加强公租房和小区物业管理服务。加快建立多主体供给、多渠道保障、租购并举的住房制度，构建房地产市场健康发展长效机制。

五、突出基础设施建设，不断提升城乡发展水平

着眼长远，破解发展瓶颈，夯实发展基础。高水平抓好城镇建设。统筹做好新一轮城市总体规划、国土空间规划和土地利用总体规划编制工作，开展闲置土地清理专项行动，提高土地利用效率。深入实施东接东进战略，加快融入长株潭一体化发展。落实《益沅桃城镇群城镇体系规划（2016－2030年）》和基础设施建设计划，加大益沅桃城镇群建设力度。加快推进一园两中心、资江风貌带建设。实施新型智慧城市建设年度重点项目。改善城区路网结构，完成康富路、文昌路、贺家桥路等道路提质改造，加快推进城市西环线、关山路、团圆南路建设。完善公共交通体系。抓好海绵城市建设，因地制宜推进新老城区雨污分流和城市“双修”，继续实施“三微”项目。强力推进“铁锤行动”，坚决遏制新增违建，加快消除存量违建，健全长效管控机制。深化城管体制改革，完善路（巷）长制，提升管理科学化、精细化、智能化水平。加强县城和重点镇建设，推广新型城镇化省级试点经验，培育一批特色小镇。力争全市城镇化率达到53%以上。继续实施交通建设大会战。完成交通建设投资80亿元。全力支持常益长高铁建设，编制“高铁新城”片区规划，启动高铁站房和路网建设。加快推进长益复线、官新、宁韶、伍益高速公路建设，确保南益高速全线建成通车。加快推进S238东坪至渠江、G234长春至谢林港、S322漉湖至乐园、S221大通湖互通至河坝、G207桃江武潭改线工程等干线公路建设。完成农村公路建设和提质改造915公里。不断完善水利信息能源基础设施。完成水利建设投资25亿元。加快兰溪河引水工程建设，完成五七运河、沱江、大通湖垸东南片补水工程。抓好堤防水库除险加固、中小河流治理、小型农田水利、沟渠塘坝清淤增蓄等项目建设，强化质量监督管理。实施农村安全饮水巩固提升工程，创新运行管理模式。做好赫山、安化国家气象观测站迁建工作。实施通信基础设施能力提升行动，推进电信普遍服务和5G网络建设。完善供气设施，推动城东天然气站建设。全力实施电网供电能力提升三年行动计划，加速推进电网改造升级。支持发展新型清洁能源，加快热电联产集中供热项目建设。

六、深化改革开放，持续激发发展动能

深入实施创新引领开放崛起战略，坚持解放思想不停步，坚持改革开放不动摇，不断激发高质量发展活力。全面深化各项改革。以机构改革为重点，完成年度各项改革任务。深化“放管服”改革，持续精简审批事项、优化审批流程、压缩审批时限。深化财税改革，强化税收协控联管，提升征管质效。加大教育、国资、农村土地制度、市场监管等领域改革力度，推动改革成果全民共享。积极推进创新发展。严格执行促进科技成果转化“一法一办法”，落实推进科技创新各项政策，扩大全社会研发经费投入。新增高新技术企业20家以上。强化企业创新主体作用，鼓励企业设立研发准备金，推动产学研用紧密结合，力促专利技术成果转化。指导帮助企业申报科技项目，争取更多项目纳入国省计划。加强知识产权保护，促进“双打”工作规范化、科学化。加大人才引进培养力度，实施更加积极灵活的人才政策，营造尊重人才的良好环境。着力提升开放实效。坚持走开放崛起之路，高水平引进来，大踏步走出去，提高对外开放度。加强与国外友好城市交流合作，推动中以合作示范城市建设。深入推进“五大开放行动”，主动对接国家大战略，对接500强，对接北上广，着力开展精准招商，引进一批发展前景好、投资规模大、带动能力强的好项目、大项目，着力抓好重大签约项目跟踪服务，切实提高履约率。精心承办2019年湖南经济合作洽谈会暨第九届湘商大会，充分发挥“汇智聚力·建设益阳”恳谈会平台作用，吸引湘商来益、益商回归。依托益阳海关等外贸服务机构，大力实施“破零倍增”计划，引导企业参与“湘品出湘”“湘企出海”行动，推动对外贸易取得新突破。

七、强化民生保障，切实维护社会和谐稳定

践行以人民为中心的发展思想，提升人民群众的获得感、幸福感、安全感，让发展更有温度、更有质感。把稳就业摆在突出位置。统筹抓好高校毕业生、农村转移劳动力、城镇困难人员、去产能职工等群体就业，拓宽就业渠道，保持就业帮扶动态“双清零”。加强职业技能培训，建立农村劳务经纪人制度。开展创业服务能力提升行动，推进创业孵化基地建设，鼓励支持各类群体自主创业、带动就业。加强社会保障体系建设。推进养老保险制度改革，落实阶段性降低社保费率政策，开展失业保险援企稳岗“护航行动”，减轻企业参保负担。完善基本医疗保险异地直接结算机制。健全社会救助体系，精准救助农村低保和社保兜底扶贫对象，做到应救尽救、应养尽养。落实军人优待政策，维护军人军属合法权益。做好城镇困难群体脱困和移民避险解困工作，关爱留守儿童、失独家庭、独居老人等特殊群体。加强残疾康复服务，发展残疾人事业。推进生命文化园建设，积极探索乡村公墓建设模式。加大保障性住房建设力度，加快推进棚户区改造。发展公平而有质量的教育。贯彻全国全省教育大会精神，坚持教育优先发展，切实加大教育投入，全面落实立德树人根本任务。加快公办幼儿园建设，扩大学位供给，促进学前教育健康发展。推动城乡义务教育一体化，合理统筹公办民办义务教育协调发展。加快中心城区“四建五促十二扩”学校建设，完成消除大班额年度任务。切实改善高中学校办学条件，鼓励高中教育优质多样发展。支持职业教育产教融合和高校“双一流”建设。全面深化教师队伍建设改革，加大定向师范生培养力度。开展校外培训专项整治行动，全面强化校车安全、校园及周边环境综合治理、食品安全、疾病防控等安全管理，让孩子安心、让家长放心。推进“健康益阳”建设。深化公立医院改革，加强医联体建设，完善“三医联动”机制，推进公立医院薪酬制度改革试点工作。加强妇幼保健服务，严格控制出生缺陷发生率。深入开展爱国卫生运动，强化传染病监测预警和联防联控。加强县级医院综合能力、基层医疗机构和全科医生队伍建设，实施远程诊疗和家庭医生签约服务，减轻群众就医负担。落实“四个最严”要求，强化食品药品质量监管，确保人民群众“舌尖上的安全”。积极发展文化体育事业。完善公共文化服务体系，抓好基层综合性文化服务中心标准化建设，推进群众文化权益均等化。挖掘文化资源，创新文化产品，发展文化产业。办好第八届花鼓戏汇演，做好第八批国家级文物保护单位申报工作。积极创建全民运动健身模范市，承办中国新能源汽车拉力锦标赛等体育赛事，着力建设“运动益阳”。提升应急管理水平。建立健全应急管理体制机制，加强应急救援指挥平台和应急救援队伍建设。持续推动消防基础建设，增强城市防灾减灾能力。深入开展安全生产“两大主题活动”，全面提升安全监管能力，强化企业主体责任，坚决遏制安全生产事故，确保人民群众生命财产安全。维护社会大局稳定。纵深推进扫黑除恶专项斗争，依法严惩各类黑恶势力，坚决斩断利益链、冲破关系网、打掉保护伞。深入开展平安创建，推进雪亮工程、一村一辅警、城市快警建设。保持对毒品犯罪高压严打态势，全力以赴打好新一轮禁毒人民战争。创新发展新时代“枫桥经验”，健全社会矛盾纠纷预防排查化解管控机制。深化信访制度改革，压实信访工作责任，推动依法就地解决合法诉求。健全公共法律服务体系，加强法律援助，维护困难弱势群体合法权益。落实“谁执法谁普法”责任制，加大普法力度。依法管理民族宗教事务，推动民族乡村经济社会发展。健全守信联合激励和失信联合惩戒机制，做到“一处失信、处处受限”，建设诚信益阳。深入推进全国文明城市创建，常态开展“六大专项整治”行动，提升市民素质，倡树文明新风，促进社会和谐。

切实抓好国防动员、国防教育、兵役征集、“双带双促”、人民防空和军民融合深度发展工作。落实意识形态工作责任制，支持主流媒体做优做强。继续抓好第四次经济普查、第三次国土调查和第二次全国污染源普查。支持工会、共青团、妇联等群团组织开展工作。统筹推进国家安全、住房公积金、老龄、援疆、地方志、档案、保密、地震、气象、水文等各项工作。

八、加强自身建设，努力打造高效法治廉洁政府

进一步解放思想、转变职能、优化服务，推动治理体系和治理能力现代化。着力建设高效政府。大兴学习之风、调查研究之风，努力提升为民服务的能力和水平。把人民群众满意作为政务服务的第一标准，增强责任意识、担当意识、作为意识，雷厉风行、“马上就办”，不断提高办事效率。着力优化政务环境，坚决打通“中梗阻”，切实做到为项目建设和企业发展只设“路标”、不设“路障”。进一步规范检查考核工作，减轻基层负担，让大家有更多时间抓工作落实。完成市县两级机构改革，构建系统完备、科学规范、运行高效的机构职能体系。加强政务服务场所建设，落实“一门办理”要求，推动系统互通、数据共享，全面落实“马上办网上办就近办一次办”要求，确保“互联网+政务服务”持续走在全省前列。着力建设法治政府。自觉接受人大及其常委会法律监督和工作监督，主动接受政协民主监督，高质量办理人大代表建议和政协委员提案，虚心听取和采纳各民主党派、工商联和无党派人士意见建议，重视舆论监督和社会监督，让权力始终在阳光下运行。支持人大立法，落实地方法规。支持检察机关开展公益诉讼。推进依法行政，健全科学决策机制，强化行政行为监督，加强合法性审查，防范行政决策风险，确保政府工作经得起历史和人民的检验。着力建设廉洁政府。履行全面从严治党主体责任和党风廉政建设“一岗双责”，坚持把政治纪律和政治规矩挺在前面，严格落实中央八项规定，以永远在路上的恒心和韧劲推进正风肃纪。着力做好巡视巡察整改后半篇文章，切实将权力关进制度的笼子，确保权力运行不越位、不错位、不空位。强化审计监督，加强财政资金支出、工程项目建设、公共资源交易等重点领域廉政风险防控体系建设，堵住廉政风险漏洞。严厉惩治各类腐败行为，坚持有腐必反、有贪必肃，坚决铲除滋生腐败的土壤，坚决消除权力寻租的空间，坚决斩断伸向群众利益的“黑手”。着力构建“亲”“清”政商关系，以重商的自觉、亲商的底气、安商的情怀，把益阳这方山水流淌着的理性平和、开放包容的人文血脉，转化为优化营商环境的人文魅力，以为民务实清廉的作风赢得人民群众的信任和支持，汇聚起推动益阳高质量发展的磅礴力量。

各位代表，时代是出卷人，我们是答卷人。不忘初心，方能回答时代之问；牢记使命，才能交出满意答卷。让我们紧密团结在以习近平同志为核心的党中央周围，高举习近平新时代中国特色社会主义思想伟大旗帜，在省委、省政府和市委的坚强领导下，紧紧依靠全市人民，只争朝夕、锐意进取，以优异成绩庆祝中华人民共和国成立70周年！
